# Bridging the gaps among research, policy and practice in ten low- and middle-income countries: Development and testing of a questionnaire for health-care providers

**DOI:** 10.1186/1478-4505-8-3

**Published:** 2010-01-29

**Authors:** G Emmanuel Guindon, John N Lavis, Boungnong Boupha, Guang Shi, Mintou Sidibe, Botagoz Turdaliyeva, Research to Policy and Practice Study Team (RPPST)

**Affiliations:** 1 Centre for Health Economics and Policy Analysis, McMaster University, Hamilton, Ontario, Canada; 2 Department of Clinical Epidemiology and Biostatistics, McMaster University, Hamilton, Ontario, Canada; 3 Department of Political Science, McMaster University, Hamilton, Ontario, Canada; 4 McMaster Health Forum, Hamilton, Ontario, Canada; 5 National Institute of Public Health, Vientiane, Lao People's Democratic Republic; 6 Council of Medical Sciences, Ministry of Health, Vientiane, Lao People's Democratic Republic; 7 Department of Health Policy and Regulation, Ministry of Health, Beijing, PR China; 8 Direction des Études de la Recherche et de la Formation, Comité National d' Éthique, Dakar, Senegal; 9 Department of Health Policy and Management, Kazakh National Medical University, Almaty, Kazakhstan; 10 Evidence-Based Health Centre, Almaty, Kazakhstan

## Abstract

**Background:**

The reliability and validity of instruments used to survey health-care providers' views about and experiences with research evidence have seldom been examined.

**Methods:**

Country teams from ten low- and middle-income countries (China, Ghana, India, Iran, Kazakhstan, Laos, Mexico, Pakistan, Senegal and Tanzania) participated in the development, translation, pilot-testing and administration of a questionnaire designed to measure health-care providers' views and activities related to improving their clinical practice and their awareness of, access to and use of research evidence, as well as changes in their clinical practice that they attribute to particular sources of research evidence that they have used. We use internal consistency as a measure of the questionnaire's reliability and, whenever possible, we use explanatory factor analyses to assess the degree to which questions that pertain to a single domain actually address common themes. We assess the questionnaire's face validity and content validity and, to a lesser extent, we also explore its criterion validity.

**Results:**

The questionnaire has high internal consistency, with Cronbach's alphas between 0.7 and 0.9 for 16 of 20 domains and sub-domains (identified by factor analyses). Cronbach's alphas are greater than 0.9 for two domains, suggesting some item redundancy. Pre- and post-field work assessments indicate the questionnaire has good face validity and content validity. Our limited assessment of criterion validity shows weak but statistically significant associations between the general influence of research evidence among providers and more specific measures of providers' change in approach to preventing or treating a clinical condition.

**Conclusion:**

Our analysis points to a number of strengths of the questionnaire - high internal consistency (reliability) and good face and content validity - but also to areas where it can be shortened without losing important conceptual domains.

## Introduction

As part of a larger project that sought to explore the factors that explain whether and how the producers and users of research support the use of and/or use research evidence as inputs to decision making, a survey was conducted of health-care providers practicing in one of four areas relevant to the Millennium Development Goals (prevention of malaria, care of women seeking contraception, care of children with diarrhoea and care of patients with tuberculosis) in each of China, Ghana, India, Iran, Laos, Kazakhstan, Mexico, Pakistan, Senegal and Tanzania. Within each health topic, a particular emphasis was placed on an intervention that was supported by strong international and local research evidence: (1) insecticide-treated materials (ITMs) to prevent malaria; (2) intrauterine devices (IUDs) for family planning; (3) oral rehydration therapy (ORT) to prevent dehydration in children with diarrhoea; and (4) DOTS strategy to control tuberculosis. The survey sought to examine health-care providers' views and activities related to improving their clinical practice and their awareness of, access to and use of research evidence, as well as changes in their clinical practice that they attribute to particular sources of research evidence that they have used.

This article focuses on the development and the reliability and validity testing of the data collection instrument. The reliability and validity of instruments used to survey health-care providers' views about and experiences with research evidence have seldom been examined. A questionnaire used to examine barriers to research utilization in nursing is a notable exception[1-3]. To our knowledge, this is the first attempt to document the development and testing of an instrument that seeks to examine comprehensively health-care providers' views about and experiences with research evidence, particularly health-care providers in low- and middle-income countries. We describe elsewhere the findings from our survey of providers, as well as the first phase of the study, which involved surveying researchers who conducted research related to one of the four topics[4-6].

## Methods

### Questionnaire development

#### Conceptual domains and question/item selection

In 2002-3 a preliminary study conducted in four of the ten countries (China, Ghana, India and Mexico) started the process of identification of relevant conceptual domains and questions. From 2003 it was taken forward using an organizing framework drawn from the framework for capacities to bridge the gap between research and action described by the Canadian Health Services Research Foundation (CHSRF)[7]. The CHSRF framework covers four dimensions of capacity, namely the capacity to acquire, assess, adapt and apply research evidence. A greater emphasis was placed on possible measures of providers' ability to acquire and apply research evidence. Relevant conceptual domains were selected for inclusion through an extensive consultation process involving all ten country teams and relevant content experts and a follow-up workshop that brought together content experts and pilot-country investigators also informed the identification of relevant conceptual domains. The following conceptual domains were identified:

- Access to information technology (IT)

- Awareness, access and use of *electronic/online *and *paper *sources of information

- Perceived influence of *electronic/online *and *paper *sources of information on clinical practice

- Training and unmet training needs

- Trust in types of sources of information (hierarchy of evidence for questions about effects)

- Extent of research utilization

- Attitudes to issues needed to be addressed to improve practice

- Activities to improve practice (such as interaction with researchers, patient groups, NGOs, for-profit organizations, policy-makers and peers)

- Attitudes and perceptions to where research is performed and reported

- Knowledge and practices specific to providers' clinical domains

Questions related to health-care providers' views and activities, their awareness of, access to and use of research evidence, and changes in their clinical practice were drawn and adapted from existing sources [8-16]. The final version of the questionnaire contained 16 questions (116 specific items) related to the aforementioned conceptual domains and an additional 14 questions related to health-care providers' characteristics. The questionnaire was finalized in October 2004 (see Additional file 1).

#### Response scales

For the most part, the original response scale was kept unchanged or only slightly adapted, most often to increase reliability by increasing the number of response options[17]. Following Wilson et al.,[14] access to information technology was measured using a 4-point ordinal scale: *easy access, less easy access, not easy access, no access*. The following definitions were provided: "easy access" is defined as either in your office or consulting room or in another part of the facility in which you work; "less easy access" is defined as shared with other staff; and "not easy access" is defined as not in the facility in which you work. Awareness of, access to and use of sources of specific information sources was adapted from Wilson et al.,[13] and was measured using a 6-point ordinal scale: *unaware, aware but not accessible, accessible but never used, used/read 3-4 times or less often per year, used/read about once a month, used/read weekly or more often.* Trust in types of sources of information was measured using a 5-point ordinal scale: *do not trust at all, distrust somewhat, neither trust nor distrust, trust somewhat, trust completely, don't know*[15]. For questions that asked about the frequency with which a health-care provider undertook certain activities, a simple 5-point ordinal scale was used: *never, rarely, sometimes, often, very often, not applicable*. Similarly, for questions which measured attitudes, various 5-point ordinal scales were used: *unimportant, somewhat important, moderately important, important, very important; very unlikely, unlikely, neutral, likely, very likely*; and, *extremely poor, below average, average, above average, excellent*. Dichotomous response scales (yes/no) were used to measure perceived influence of *electronic/online *and *paper *sources of information on clinical practice, training, unmet training needs and interaction with researchers, patient groups, non-governmental organizations (NGOs), for-profit organizations, policy-makers and peers.

#### Translation

For the six countries in which English is not spoken widely (China, Iran, Kazakhstan, Laos, Mexico and Senegal), the questionnaire was translated by the World Health Organization's (WHO) translation service (Mandarin, French, Russian and Spanish) and country teams (Lao and Persian). Country teams assessed the quality of the translation, made minor wording corrections when required, piloted the draft questionnaire, and made additional minor wording corrections when required.

### Sample and questionnaire administration

Resource constraints prevented the survey of a fully representative sample of providers in all study sites. The country teams sought to survey at least 100 health-care providers for each topic in each country. The sampling frame was developed by each country using lists of health-care providers identified by country investigators. The country teams employed several approaches to increase the response rate: personalized letters, follow up of contacts and providing a set of WHO publications as an incentive [18]. Random sampling processes were used in all countries except Tanzania which used a purposive approach to sample district medical officers and Kazakhstan where the whole population of gynecologists in Almaty city was sampled. Before data collection began, the questionnaire was piloted in all countries by country teams and by the central team among WHO staff in Geneva, Switzerland. Data collection took place between late 2004 and the end of 2005. (A detailed description of the sampling designs for each country/topic combination is provided in Additional file 2).

### Reliability

The reliability of an instrument is the extent to which it measures the conceptual domains in a reproducible fashion[17]. We use internal consistency to assess reliability. Internal consistency measures the extent to which items that attempt to measure a single conceptual domain provide consistent responses. We use Cronbach's alpha, a measure of internal consistency based on correlation between items measuring a single conceptual domain. Moderate to high correlation among items provide a balanced approach to item selection. Streiner and Norman[17] recommend alpha of at least 0.70 but no higher than 0.90. A very large alpha may indicate a high level of item redundancy.

Whenever possible, we use explanatory factor analysis to assess the degree to which questions that pertain to a single domain address common themes. We use factor analysis specifically to explore the relationship among items within each of the five conceptual domains that used an ordinal response scale, and hence the possibility of reducing the number of items. Before performing factor analysis, we examine if the sample has a suitable factorial structure. We use two formal statistical tests: the Bartlett Test of Sphericity and the Kaiser-Meyer-Olkin Measure of Sampling Adequacy. We use the iterated principal-factor method (IPF) to analyze the correlation matrix, which re-estimates the communalities iteratively. (The communality measures the percent of variance in a given variable explained by all the factors jointly). The number of factors included in the analysis is chosen using the Kaiser criterion (i.e. if factors' eigenvalue (a measure of how much variation is explained by the factor) exceed 1.0) and Cattell's Scree Test[19]. To help with the interpretability of the factors, we use orthogonal (varimax) and oblique (promax) methods of rotation.

As an indirect check on whether the grouping of conceptually related items may have contributed to a pattern of identical responses, we calculated the proportion of respondents who provided the same ordinal scale response for each item within a conceptual domain (e.g. always chose the response "trust somewhat" when asked about trust in different sources of information).

### Validity

An instrument is valid if it accurately reflects the conceptual domains it is designed to measure. We assess the questionnaire in terms of its face validity and content validity. To a lesser extent, we also explore its criterion validity. Face validity is a simple indication that, on the face of it, the instrument appears to be measuring the desired conceptual domains. Similarly, content validity indicates whether the instrument attempts to measure all the relevant and important domains. Assessing criterion validity involves correlating a scale with a criterion measure[17].

## Results

A total of 1,629 health-care providers were sampled in the ten countries, of which 1,499 completed and returned the questionnaire, resulting in an overall response rate of 92%. The number of health-care providers sampled varied across country/topic (100 to 140) as did the response rates (0.44 to 1.00). (Detailed response rates for each country/topic combination are provided in Additional file 2, Table S1).

### Internal consistency

Cronbach's alphas are between 0.7 and 0.9 for 16 of 20 domains and sub-domains (identified by factor analyses), indicating good internal consistency (Table 1). Cronbach's alphas were greater than 0.9 for two domains (the extent of research utilization and awareness, access and use of electronic/online of information). Such high Cronbach's alphas suggest some item redundancy.

**Table 1:** Assessments of internal consistency

Conceptual domains	# of items	Cronbach's Alpha	Proportion who provided the same ordinal scale response for each item within a conceptual domain
**Access to information technology (IT)**			

-Personal computer without a CD ROM; Personal computer with a CD ROM; Internet	3	0.867	0.707

**Awareness, access and use of *electronic/online *and *paper *sources of information**			

-*Electronic/online*: Medical textbooks; Clinical practice guidelines; DARE; Cochrane Library; Open access initiatives (HINARI, other); Bibliographic databases (international, regional), Scientific journals (from high-income countries, own region, own country); Articles, reports, reviews (from public and not-for-profit health organizations, for-profit health organizations); Summaries of articles, reports, and reviews from public and not-for-profit health organizations.	14	0.908	0.055

*Factor 1*. DARE; Cochrane Library; Open access initiatives (HINARI, other); Bibliographic databases (international, regional), Scientific journals (from high-income countries)	7	0.854	0.121

*Factor 2*. Medical textbooks; Clinical practice guidelines; Scientific journals from own country; Articles, reports, reviews (from public and not-for-profit health organizations, for-profit health organizations); Summaries of articles, reports, and reviews from public and not-for-profit health organizations.	6	0.880	0.072

-*Paper*: Medical textbooks; Clinical practice guidelines; Scientific journals (from high-income countries, own region, own country); Articles, reports, reviews (from public and not-for-profit health organizations, for-profit health organizations); Summaries of articles, reports, and reviews from public and not-for-profit health organizations.	8	0.823	0.057

**Perceived influence of *electronic/online *and *paper *sources of information on clinical practice** ***[Changed approach to preventing or treating a clinical condition]***			

-*Electronic/online*: Medical textbooks; Clinical practice guidelines; DARE; Cochrane Library; Open access initiatives (HINARI, other); Bibliographic databases (international, regional), Scientific journals (from high-income countries, own region, own country); Articles, reports, reviews (from public and not-for-profit health organizations, for-profit health organizations); Summaries of articles, reports, and reviews from public and not-for-profit health organizations.	14	0.845	n/a

-*Paper*: Medical textbooks; Clinical practice guidelines; Scientific journals (from high-income countries, own region, own country); Articles, reports, reviews (from public and not-for-profit health organizations, for-profit health organizations); Summaries of articles, reports, and reviews from public and not-for-profit health organizations.	8	0.805	n/a

**Training**			

-General computer skills; Searching the internet; Acquiring systematic reviews through the Cochrane Library; Acquiring copies of full-text journal articles from open access initiatives; Acquiring titles and abstracts of articles from bibliographic databases; Critically appraising clinical practice guidelines, clinical protocols, and/or clinical decision support tools; Critically appraising systematic reviews; Critically appraising individual studies of a diagnostic tool and/or approach; Critically appraising individual studies of the effectiveness of an intervention; Critically appraising economic evaluations; Adapting research evidence to local settings.	11	0.898	0.454

**Trust in types of sources of information**			

-Single cohort study; Systematic review of randomized controlled double-blind trials; Practical experience; Single case control study; Single randomized controlled double-blind trials; Case series; Expert opinion and advice; Case report.	8	0.830	0.075

*Factor 1*. Single cohort study; Systematic review of randomized controlled double-blind trials; Single case control study; Single randomized controlled double-blind trial.	4	0.815	0.252

*Factor 2*. Practical experience; Case series; Expert opinion and advice; Case report.	4	0.788	0.176

**Extent of research utilization**			

-Reception; Cognition; Discussion; Reference; Adoption; Influence.	6	0.929	0.173

**Attitudes to issues needed to be addressed to improve practice**			

-Financial incentives (e.g., better pay); More staff; More training; More feedback on staff performance; More/better equipment or supplies; Better security; Better physical environment; Higher quality of available research; More access to peers/networks; More locally applicable research	10	0.827	0.100

*Factor 1*. Financial incentives (e.g., better pay); More staff; More training; More feedback on staff performance; More/better equipment or supplies.	5	0.735	0.192

*Factor 2*. Higher quality of available research; More access to peers/networks; More locally applicable research.	3	0.798	0.464

*Factor 3*. Better security; Better physical environment.	2	0.688	0.602

**Activities to improve practice**			

-Working with: researchers or researcher groups; patient groups; representatives of non-governmental organizations (NGOs); representatives of for-profit organizations; policy-makers; peers.	6	0.701	0.147

**Attitudes and perceptions to where research is performed and reported**			

-High-income countries	4	0.734	0.132

-Own region	4	0.517	0.186

-Own country	4	0.724	0.194

Note: n/a: not applicable			

Factor analyses satisfied the sample adequacy criteria, with most values above 0.80 and the null hypothesis of uncorrelated variables of the Bartlett Test of Sphericity rejected at the 1 percent level in all cases. (Results of the diagnostic checks and the factor analyses are presented in Additional file 3). The results for the five factor analyses are as follows.

#### 1) Awareness, access and use of electronic/online sources of information

The Kaiser criterion and Cattell's Scree Test both suggest including the same number of factors. Two factors were identified with eigenvalues (i.e. the variance of the factor) of 6.16 and 1.37. Interestingly, 'academically oriented' information sources tend to load together in factor 1 (DARE, Cochrane Library, open access initiatives, bibliographic databases and scientific journals from high-income countries). Factor 2 groups medical textbooks, clinical practice guidelines, and scientific journals from their own country, along with articles, reports and reviews from public/not-for-profit and for-profit health organizations. Scientific journals from their own region is found to be factorially complex (i.e. it loads on both factors to a comparable degree).

#### 2) Awareness, access and use of paper sources of information

The Kaiser criterion suggests this conceptual domain may be one-dimensional while Cattell's Scree Test suggests including two factors with eigenvalues of 3.26 and 0.78. Both rotated solutions find three items (scientific journals from high-income countries, their own region, and their own country) to be factorially complex. In addition, only two items (medical textbooks, and clinical practice guidelines) load on factor 2. On the whole, there is little to suggest this conceptual domain is not one-dimensional.

#### 3) Trust in types of sources of information (hierarchy of evidence)

The Kaiser criterion and Cattell's Scree Test both suggest including the same number of factors. Two factors were identified with eigenvalues of 3.38 and 1.10. Both rotated solutions suggest clear distinct loadings based on the hierarchy of evidence for questions about effects (systematic review of RCTs, RCT, cohort study and case control study vs. case series, case report, expert opinion and advice and practical experience)[20].

#### 4) Extent of research utilization

Only one factor was identified (eigenvalue = 4.23) indicating this particular conceptual domain is one-dimensional.

#### 5) Attitudes to issues needed to be addressed to improve practice

The Kaiser criterion suggests this conceptual domain may be one-dimensional while Cattell's Scree Test suggests including three factors with eigenvalues of 3.72, 0.83 and 0.55. We explore the rotated solutions while retaining three and two factors. The solution with three factors is more intuitive. Factor 1 groups financial incentives, more staff, more training and more feedback on staff performance. Factor 2 groups higher quality of available research, more access to peers/networks and more locally applicable research. Factor 3 groups better security and better physical environment.

The analysis of patterns of identical responses suggests that some identical pattern response (i.e. respondents who provided the same ordinal scale response for each item within a conceptual domain) may exist for two composite questions: access to information technology (IT) (0.71) and training (0.45). These results, however, are unlikely to be problematic as they are driven by respondents who reported having no access to IT (0.58) and not having received any training since completing their last degree (0.44). Within sub-domains identified through factor analyses, the frequency of identical responses is relatively high for two composite questions: attitudes to issues needed to be addressed to improve practice and trust in types of sources of information.

### Validity

Face validity and content validity were assessed first by all country teams and second by technical experts with international and/or local experience that possessed expertise related either to supporting the use of research evidence in clinical practice or to the particular health topics being examined. Prior to beginning data collection, the questionnaire was pilot tested in all sites among at least five health-care providers and/or clinical researchers. Additionally, a post-field workshop was convened in July 2005 and was attended by nine of ten country team representatives (a representative from Tanzania was unable to attend, but did participate in post-workshop teleconference), along with WHO staff, McMaster University researchers, and representatives of the Council on Health Research for Development (COHRED).

We explored criterion validity for the measure of the extent of research utilization, a general measure of the impact of research use often utilized in the literature,[8] by examining Spearman's rank correlation between its *influence *dimension and more specific measures of change in approach to preventing or treating a clinical condition. We find weak but statistically significant positive correlations.

## Discussion

### Principal findings

The *WHO/McMaster Questionnaire on Providers' use of Research Evidence *has acceptable levels of internal consistency for 16 of 20 domains and sub-domains (identified by factor analyses). The questionnaire has high Cronbach's alphas (> 0.90) for two domains, suggesting some item redundancy. Consequently pairs of items (DARE/Cochrane Library, HINARI/other open access initiatives, and international/regional bibliographic databases) can be reduced in number. Also, the first five items (reception, cognition, discussion, reference, and adoption) in Landry's *extent of research utilization *scale (based on the work of Knott and Wildavsky [21]) can conceivably be omitted. However, given the possibility that linguistic or cultural differences may have affected providers' interpretation of these items, additional research is needed before recommending that these items be omitted. Pre- and post-fieldwork assessments indicate the questionnaire has good face validity and content validity. Our limited assessment of criterion validity shows weak but statistically significant associations between the general influence of research evidence among providers and more specific measures of providers' change in approach to preventing or treating a clinical condition.

### Strengths and limitations of the study

The development and testing of the questionnaire had a number of strengths: 1) the development process drew on a well established organizing framework, a thorough literature review, an extensive consultation process with a broad range of researchers in ten low- and middle-income countries and with a diverse array of content experts, and a pilot study and follow-up workshop; 2) the resulting questionnaire seeks to examine comprehensively health-care providers' views about and experiences with research evidence, particularly health-care providers in low- and middle-income countries; and 3) the validity and reliability testing process drew on data and experiences from ten low- and middle-income countries and this process suggests that the questionnaire has good internal consistency (reliability), face validity, content validity, and (while based on a limited assessment) criterion validity.

The weaknesses in the development and testing of the questionnaire are as follows: 1) resource constraints did not allow us to examine the questionnaire's test-retest reliability, which is a measure of its repeatability during an interval when no change in respondent's activities and attitude is expected,[17] and we encourage anyone interested in using the questionnaire to do so; and 2) we did not examine fully the questionnaire's criterion validity (we used as a criterion additional self-reported data given that no external reference standard exists) and we encourage anyone interested in using the questionnaire to use a qualitative approach (e.g. case studies) to examine whether and how self reports accord with actual behaviours. That said, the low reported frequencies of some behaviors (e.g. use of the Cochrane Library) that are widely believed to be beneficial suggest that the social desirability bias is not pervasive.

## Conclusion

Our analysis points to a number of strengths of the questionnaire - high internal consistency (reliability) and good face and content validity - but also to areas where it can be shortened without losing important conceptual domains. Moreover, the questionnaire can easily be adapted and used to examine providers' views about and experiences with research evidence on any clinical topic. Future users of the questionnaire are advised to further examine elements of its reliability and validity.

## Competing interests

The authors declare that they have no competing interests.

## Authors' contributions

GEG 1) contributed substantially to conception and design, acquisition of data, and analysis and interpretation of data, 2) drafted the article and revised it critically for important intellectual content, and 3) gave final approval of the version to be published. JNL, BB, GS, MS, and BT: 1) contributed substantially to conception and design, acquisition of data, and/or analysis and interpretation of data, 2) provided feedback on the article; and 3) gave final approval of the version to be published. Members of the Research to Policy and Practice Study Team: 1) contributed substantially to acquisition of data; 2) reviewed drafts of the article; and 3) gave final approval of the version to be published.

## Supplementary Material

Additional file 1 World Health Organization/McMaster University Questionnaire on Research Use in the Health Sector. Click here for file

Additional file 2 **Sample**. Table S1: Sample sizes and response rates by country and health topics. Table S2: Sampling design by country and health topics. Click here for file

Additional file 3 **Factor analyses**. Additional file 3.1: Factor analyses: Awareness, access and use of electronic/online sources of information. Table S3.1-1, Table S3.1-2 and Table S3.1-3.
Additional file 3.2: Factor analyses: Awareness, access and use of paper sources of information. Table S3.2-1, Table S3.2-2: and Table S3.2-3.
Additional file 3.3: Factor analyses: Trust in types of sources of information. Table S3.3-1, Table S3.3-2 and Table S3.3-3.
Additional file 3.4: Factor analyses: Extent of research utilization. Table S3.4-1.
Additional file 3.5: Factor analyses: Attitudes to issues needed to be addressed to improve practice. Table S3.5-1, Table S3.5-2, Table S3.5-3, Table S3.5-4 and Table S3.5-5. Click here for file
