# Supplementary material for: Bridging the gaps among research, policy and practice in ten low- and middle-income countries: Development and testing of a questionnaire for health-care providers
Source: Health Res Policy Syst. 2010 Jan 29;8:3. doi: 10.1186/1478-4505-8-3 (PMC2825186; doi:10.1186/1478-4505-8-3)
Supplement: Additional file 1 — World Health Organization/McMaster University Questionnaire on Research Use in the Health Sector. [file 1478-4505-8-3-S1.DOC]

| There is currently a large volume of research evidence relevant to the delivery of health-care services (e.g., effectiveness and cost-effectiveness of preventive, diagnostic and treatment interventions) on which health-care providers can draw to inform their everyday practice.  The purpose of this questionnaire is to learn more about how you acquire, assess, adapt and apply research evidence and the context in which you work. Completing this questionnaire indicates that you have read and understood the information regarding participation in the research project and agree to participate.  Please check or circle the most appropriate response for each question. If you have specific comments on any issues raised in particular questions, please identify the question by number and add your comments in the space provided on the final page of the questionnaire.  Please note that for consistency we use the term “facility” throughout this questionnaire to refer to your work setting (e.g., community health centre, hospital, private clinic, etc.). If you work in more than one facility, please answer all questions while keeping in mind *all* facilities in which you work. For example, if you were asked whether you have access to the internet and you have access to the internet in your private office but not in the hospital at which you also work, please answer that you have access to the internet. |
| --- |

i. Please indicate whether you are currently involved in the [prevention of malaria/care of patients with tuberculosis/care of children with diarrhoea/care of women seeking contraception]. [ONLY ONE HEALTH TOPIC TO BE INCLUDED]

❑Yes (Please continue.)

❑ No (Please return the questionnaire with only this box ticked.)

1. Please indicate whether you (or someone who can act on your behalf) had access to any of the following over the past 12 months. Please note the following definitions: "easy access" is defined as either in your office or consulting room or in another part of the facility in which you work; "less easy access" is defined as shared with other staff; and "not easy access" is defined as *not* in the facility in which you work.

|  | | **Yes, easy access**  **1** | **Yes, less easy access**  **2** | **Yes, but not easy access**  **3** | **No**  **Access**  **4** | **Not sure**  **0** |
| --- | --- | --- | --- | --- | --- | --- |
|  | Personal computer without a CD ROM | ❑ | ❑ | ❑ | ❑ | ❑ |
|  | Personal computer with a CD ROM | ❑ | ❑ | ❑ | ❑ | ❑ |
|  | Internet (i.e., world wide web) | ❑ | ❑ | ❑ | ❑ | ❑ |

**If you did not have access to a personal computer with a CD ROM or to the internet, please go to question 4.**

1. Please indicate whether you had access to the following *electronic/online* sources of information over the past 12 months.

| **Unaware of information source**  **1** | | **Aware of but not accessible**  **2** | **Accessible but never used/read**  **3** | **Used/read 3-4 times per year or less often**  **4** | **Used/read about once a month**  **5** | | **Used/read weekly or more often**  **6** | | | | | |
| --- | --- | --- | --- | --- | --- | --- | --- | --- | --- | --- | --- | --- |
| 1. A | Reproductive Health Library [FOR CONTRACEPTION ONLY] | | | | | 1 | | 2 | 3 | 4 | 5 | 6 |
|  | Medical textbooks (e.g., Users’ Guides to the Medical Literature, Scientific American Medicine) | | | | | 1 | | 2 | 3 | 4 | 5 | 6 |
|  | Clinical practice guidelines, clinical protocols, and/or clinical decision support tools | | | | | 1 | | 2 | 3 | 4 | 5 | 6 |
|  | Database of Abstracts of Reviews of Effectiveness (DARE) | | | | | 1 | | 2 | 3 | 4 | 5 | 6 |
| 1. A | Cochrane Library (which includes both Cochrane Reviews and reviews covered in DARE) | | | | | 1 | | 2 | 3 | 4 | 5 | 6 |
|  | HINARI - Health Internetwork Access to Research Initiative (an open access initiative) | | | | | 1 | | 2 | 3 | 4 | 5 | 6 |
|  | Other open access initiatives (e.g., FreeMedicalJournals.com, African Journal Online) | | | | | 1 | | 2 | 3 | 4 | 5 | 6 |
|  | International bibliographic databases (e.g., MedLine, PubMed) | | | | | 1 | | 2 | 3 | 4 | 5 | 6 |
| 1. A | Regional bibliographic databases (e.g., LILACS, AIM, IMSEAR, IMEMR, etc.) | | | | | 1 | | 2 | 3 | 4 | 5 | 6 |
|  | Scientific journals from high-income countries (e.g., New England Journal of Medicine Online, The Lancet Online, BMJ Online) | | | | | 1 | | 2 | 3 | 4 | 5 | 6 |
|  | Scientific journals from your region (INSERT REGION NAME) (e.g., INSERT TWO REGIONAL EXAMPLES) | | | | | 1 | | 2 | 3 | 4 | 5 | 6 |
|  | Scientific journals from your country (e.g., INSERT TWO COUNTRY EXAMPLES) | | | | | 1 | | 2 | 3 | 4 | 5 | 6 |
| 1. A | Articles, reports, and reviews from public or not-for-profit health organizations such as the Ministry of Health, professional associations, non-governmental organizations and international organizations (e.g., WHO) | | | | | 1 | | 2 | 3 | 4 | 5 | 6 |
|  | Articles, reports, and reviews from for-profit health organizations (e.g., pharmaceutical companies, medical device manufacturers) | | | | | 1 | | 2 | 3 | 4 | 5 | 6 |
|  | Summaries of articles, reports, and reviews from public or not-for-profit health organizations such as the Ministry of Health, professional associations, non-governmental organizations and international organizations (e.g., WHO) | | | | | 1 | | 2 | 3 | 4 | 5 | 6 |
|  | Other electronic/online resources (please specify): ________________________________ | | | | | 1 | | 2 | 3 | 4 | 5 | 6 |
|  |  | | | | |  | |  |  | Use | | |

**If you did not have access to a personal computer with a CD ROM or to the internet, please go to question 4.**

1. Please indicate the ways in which research evidence from the following *electronic/online* sources of information influenced your clinical practice over the past 12 months. Please answer *only* for those sources of information that you used over the past 12 months (i.e. for those sources for which you circled 4, 5, or 6 in the previous question). Please circle *all* that apply.

| **Changed approach to preventing a clinical condition**  **1** | | **Changed approach to diagnosing a clinical condition**  **2** | **Changed approach to treating a clinical condition**  **3** | **Changed/developed a local clinical practice guideline, clinical protocol and/or clinical decision support tool**  **4** | **Changed the type of medication, medical devices and/or other technologies you stock in your facility**  **5** | | **Not applicable**  **na** | | | | | |
| --- | --- | --- | --- | --- | --- | --- | --- | --- | --- | --- | --- | --- |
| 1. A | Reproductive Health Library [FOR CONTRACEPTION ONLY] | | | | | 1 | | 2 | 3 | 4 | 5 | na |
|  | Medical textbooks (e.g., Users’ Guides to the Medical Literature, Scientific American Medicine) | | | | | 1 | | 2 | 3 | 4 | 5 | na |
|  | Clinical practice guidelines, clinical protocols, and/or clinical decision support tools | | | | | 1 | | 2 | 3 | 4 | 5 | na |
| 1. A | Database of Abstracts of Reviews of Effectiveness (DARE) | | | | | 1 | | 2 | 3 | 4 | 5 | na |
|  | Cochrane Library (which includes both Cochrane Reviews and reviews covered in DARE) | | | | | 1 | | 2 | 3 | 4 | 5 | na |
|  | HINARI - Health Internetwork Access to Research Initiative (an open access initiative) | | | | | 1 | | 2 | 3 | 4 | 5 | na |
| 1. A | Other open access initiatives (e.g., FreeMedicalJournals.com, African Journal Online) | | | | | 1 | | 2 | 3 | 4 | 5 | na |
|  | International bibliographic databases (e.g., MedLine, PubMed) | | | | | 1 | | 2 | 3 | 4 | 5 | na |
|  | Regional bibliographic databases (e.g., LILACS, AIM, IMSEAR, IMEMR, etc.) | | | | | 1 | | 2 | 3 | 4 | 5 | na |
| 1. A | Scientific journals from high-income countries (e.g., New England Journal of Medicine Online, The Lancet Online, BMJ Online) | | | | | 1 | | 2 | 3 | 4 | 5 | na |
|  | Scientific journals from your region (INSERT REGION NAME) (e.g., INSERT TWO REGIONAL EXAMPLES) | | | | | 1 | | 2 | 3 | 4 | 5 | na |
|  | Scientific journals from your country (e.g., INSERT TWO COUNTRY EXAMPLES) | | | | | 1 | | 2 | 3 | 4 | 5 | na |
| 1. A | Articles, reports, and reviews from public or not-for-profit health organizations such as the Ministry of Health, professional associations, non-governmental organizations and international organizations (e.g., WHO) | | | | | 1 | | 2 | 3 | 4 | 5 | na |
|  | Articles, reports, and reviews from for-profit health organizations (e.g., pharmaceutical companies, medical device manufacturers) | | | | | 1 | | 2 | 3 | 4 | 5 | na |
|  | Summaries of articles, reports, and reviews from public or not-for-profit health organizations such as the Ministry of Health, professional associations, non-governmental organizations and international organizations (e.g., WHO) | | | | | 1 | | 2 | 3 | 4 | 5 | na |
|  | Other electronic/online resources (please specify): ________________________________ | | | | | 1 | | 2 | 3 | 4 | 5 | na |

1. Please indicate whether you had access to the following *paper* sources of information over the past 12 months.

| **Unaware of information source**  **1** | | **Aware of but not accessible**  **2** | **Accessible but never used/read**  **3** | **Used/read 3-4 times per year or less often**  **4** | **Used/read about once a month**  **5** | | **Used/read weekly or more often**  **6** | | | | | |
| --- | --- | --- | --- | --- | --- | --- | --- | --- | --- | --- | --- | --- |
| 1. A | Medical textbooks (e.g., Users’ Guides to the Medical Literature, Scientific American Medicine) | | | | | 1 | | 2 | 3 | 4 | 5 | 6 |
|  | Clinical practice guidelines, clinical protocols, and/or clinical decision support tools | | | | | 1 | | 2 | 3 | 4 | 5 | 6 |
|  | Scientific journals from high-income countries (e.g., New England Journal of Medicine, The Lancet, BMJ) | | | | | 1 | | 2 | 3 | 4 | 5 | 6 |
|  | Scientific journals from your region (INSERT REGION NAME) (e.g., INSERT TWO REGIONAL EXAMPLES) | | | | | 1 | | 2 | 3 | 4 | 5 | 6 |
| 1. A | Scientific journals from your country (e.g., INSERT TWO COUNTRY EXAMPLES) | | | | | 1 | | 2 | 3 | 4 | 5 | 6 |
|  | Articles, reports, reviews from public and not-for-profit health organizations such as the Ministry of Health, professional associations, non-governmental organizations and international organizations (e.g., WHO) | | | | | 1 | | 2 | 3 | 4 | 5 | 6 |
|  | Articles, reports, and reviews from for-profit health organizations (e.g., pharmaceutical companies, medical device manufacturers) | | | | | 1 | | 2 | 3 | 4 | 5 | 6 |
|  | Summaries of articles, reports, and reviews from public and not-for-profit health organizations such as the Ministry of Health, professional associations, non-governmental organizations and international organizations (e.g., WHO) | | | | | 1 | | 2 | 3 | 4 | 5 | 6 |
| 1. A | Other paper resources (please specify): ________________________________________ | | | | | 1 | | 2 | 3 | 4 | 5 | 6 |
|  |  | | | | |  | |  |  | Use | | |

1. Please indicate the ways in which research evidence from the following *paper* sources of information influenced your clinical practice over the past 12 months. Please answer *only* for those sources of information that you used over the past 12 months (i.e. those sources for which you circled 4, 5, or 6 in the previous question). Please circle *all* that apply.

| **Changed approach to preventing a clinical condition**  **1** | | **Changed approach to diagnosing a clinical condition**  **2** | **Changed approach to treating a clinical condition**  **3** | **Changed/developed a local clinical practice guideline, clinical protocol and/or clinical decision support tool**  **4** | **Changed the type of medication, medical devices and/or other technologies you stock in your facility**  **5** | | **Not applicable**  **na** | | | | | |
| --- | --- | --- | --- | --- | --- | --- | --- | --- | --- | --- | --- | --- |
| 1. A | Medical textbooks (e.g., Users’ Guides to the Medical Literature, Scientific American Medicine) | | | | | 1 | | 2 | 3 | 4 | 5 | na |
|  | Clinical practice guidelines, clinical protocols, and/or clinical decision support tools | | | | | 1 | | 2 | 3 | 4 | 5 | na |
|  | Scientific journals from high-income countries (e.g., New England Journal of Medicine, The Lancet, BMJ) | | | | | 1 | | 2 | 3 | 4 | 5 | na |
| 1. A | Scientific journals from your region (INSERT REGION NAME) (e.g., INSERT TWO REGIONAL EXAMPLES) | | | | | 1 | | 2 | 3 | 4 | 5 | na |
|  | Scientific journals from your country (e.g., INSERT TWO COUNTRY EXAMPLES) | | | | | 1 | | 2 | 3 | 4 | 5 | na |
|  | Articles, reports, reviews from public and not-for-profit health organizations such as the Ministry of Health, professional associations, non-governmental organizations and international organizations (e.g., WHO) | | | | | 1 | | 2 | 3 | 4 | 5 | na |
| 1. A | Articles, reports, and reviews from for-profit health organizations (e.g., pharmaceutical companies, medical device manufacturers) | | | | | 1 | | 2 | 3 | 4 | 5 | na |
|  | Summaries of articles, reports, and reviews from public and not-for-profit health organizations such as the Ministry of Health, professional associations, non-governmental organizations and international organizations (e.g., WHO) | | | | | 1 | | 2 | 3 | 4 | 5 | na |
|  | Other paper resources (please specify): ________________________________________ | | | | | 1 | | 2 | 3 | 4 | 5 | na |

1. Please indicate whether you have received any training in any of the following domains since completing your last degree (e.g., MD, RN, PhD). If yes, please indicate the most recent year in which you received training. Please also indicate whether you feel you have any unmet training needs in any of the domains.

| Domains | | **Received training** | | |  | **Unmet training needs** | | |
| --- | --- | --- | --- | --- | --- | --- | --- | --- |
|  |  | **Yes**  **If so, when**  **1** | **No**  **2** | **Not sure**  **0** |  | **Yes**  **1** | **No**  **2** | **Not sure**  **0** |
|  | General computer skills | ❑ year: _____ | ❑ | ❑ |  | ❑ | ❑ | ❑ |
|  | Searching the internet | ❑ year: _____ | ❑ | ❑ |  | ❑ | ❑ | ❑ |
|  | Acquiring systematic reviews through the Cochrane Library | ❑ year: _____ | ❑ | ❑ |  | ❑ | ❑ | ❑ |
|  | Acquiring copies of full-text journal articles from open access initiatives | ❑ year: _____ | ❑ | ❑ |  | ❑ | ❑ | ❑ |
|  | Acquiring titles and abstracts of articles from bibliographic databases | ❑ year: _____ | ❑ | ❑ |  | ❑ | ❑ | ❑ |
|  | Critically appraising clinical practice guidelines, clinical protocols, and/or clinical decision support tools | ❑ year: _____ | ❑ | ❑ |  | ❑ | ❑ | ❑ |
|  | Critically appraising systematic reviews | ❑ year: _____ | ❑ | ❑ |  | ❑ | ❑ | ❑ |
|  | Critically appraising individual studies of a diagnostic tool and/or approach | ❑ year: _____ | ❑ | ❑ |  | ❑ | ❑ | ❑ |
|  | Critically appraising individual studies of the effectiveness of an intervention (e.g., randomized controlled trials) | ❑ year: _____ | ❑ | ❑ |  | ❑ | ❑ | ❑ |
|  | Critically appraising economic evaluations | ❑ year: _____ | ❑ | ❑ |  | ❑ | ❑ | ❑ |
|  | Adapting research evidence to local settings (e.g., incorporating it into a local clinical practice guideline) | ❑ year: _____ | ❑ | ❑ |  | ❑ | ❑ | ❑ |
|  | Integrated Management of Childhood  Illness (IMCI) [FOR ORT AND MALARIA ONLY] | ❑ year: _____ | ❑ | ❑ |  | ❑ | ❑ | ❑ |
|  | Care of patients with tuberculosis [FOR TB ONLY] | ❑ year: _____ | ❑ | ❑ |  | ❑ | ❑ | ❑ |
|  | Prevention of malaria [FOR MALARIA ONLY] | ❑ year: _____ | ❑ | ❑ |  | ❑ | ❑ | ❑ |
|  | Care of children with diarrhoea [FOR ORT ONLY] | ❑ year: _____ | ❑ | ❑ |  | ❑ | ❑ | ❑ |
|  | Care of women seeking contraception [FOR CONTRACEPTION ONLY] | ❑ year: _____ | ❑ | ❑ |  | ❑ | ❑ | ❑ |

1. Please indicate how much trust you have in each of the following sources of information to make clinical decisions about prevention or treatment options.

| **Do not trust at all**  **1** | | **Distrust somewhat**  **2** | **Neither trust nor distrust**  **3** | **Trust somewhat**  **4** | **Trust completely**  **5** | | | **Don’t know**  **dk** | | | | |
| --- | --- | --- | --- | --- | --- | --- | --- | --- | --- | --- | --- | --- |
|  | Single cohort study | | | | | 1 | 2 | | 3 | 4 | 5 | dk |
|  | Systematic review of randomized controlled double-blind trials | | | | | 1 | 2 | | 3 | 4 | 5 | dk |
|  | Your practical experience | | | | | 1 | 2 | | 3 | 4 | 5 | dk |
|  | Single case control study | | | | | 1 | 2 | | 3 | 4 | 5 | dk |
|  | Single randomized controlled double-blind trials | | | | | 1 | 2 | | 3 | 4 | 5 | dk |
|  | Case series | | | | | 1 | 2 | | 3 | 4 | 5 | dk |
|  | Expert opinion and advice | | | | | 1 | 2 | | 3 | 4 | 5 | dk |
|  | Case report | | | | | 1 | 2 | | 3 | 4 | 5 | dk |

1. Please indicate how often you have received research evidence about [INSERT HEALTH TOPIC], read and understood it, and used it over the past 12 months.

| **Never**  **1** | | **Rarely**  **2** | **Sometimes**  **3** | **Often**  **4** | **Very often**  **5** | | | **Not applicable**  **na** | | | | |
| --- | --- | --- | --- | --- | --- | --- | --- | --- | --- | --- | --- | --- |
|  | You have received research evidence about [INSERT HEALTH TOPIC]. | | | | | 1 | 2 | | 3 | 4 | 5 | na |
|  | You have read and understood the research evidence that you have received. | | | | | 1 | 2 | | 3 | 4 | 5 | na |
|  | You have cited research evidence to colleagues or patients as a reference in your professional practice. | | | | | 1 | 2 | | 3 | 4 | 5 | na |
|  | You have made efforts to promote the adoption of research evidence in your field of professional practice. | | | | | 1 | 2 | | 3 | 4 | 5 | na |
|  | Research evidence has led you to make professional choices and decisions that you would not have made otherwise. | | | | | 1 | 2 | | 3 | 4 | 5 | na |
|  | The utilization of research evidence has led to concrete changes in your professional practice. | | | | | 1 | 2 | | 3 | 4 | 5 | na |

The following section seeks to collect information on your current practices and views concerning [INSERT HEALTH TOPIC]

1. Please indicate how often you performed each of these practices.

[FOR ORT ONLY]

| **Never**  **1** | | **Rarely**  **2** | **Sometimes**  **3** | **Often**  **4** | **Very often**  **5** | | | **Not applicable**  **na** | | | | |
| --- | --- | --- | --- | --- | --- | --- | --- | --- | --- | --- | --- | --- |
|  | Over the past 12 months, when treating young children with moderate dehydration, how often did you treat them with an intravenous electrolyte solution? | | | | | 1 | 2 | | 3 | 4 | 5 | na |
|  | Over the past 12 months, when treating young children with mild and moderate dehydration , how often did you prescribe an antibiotic to help stop diarrhoea? | | | | | 1 | 2 | | 3 | 4 | 5 | na |
|  | Over the past 12 months, when treating infants with diarrhoea, how often did you use Oral Rehydration Salts (ORS) solution for the maintenance therapy? | | | | | 1 | 2 | | 3 | 4 | 5 | na |
|  | Over the past 12 months, when treating young children with cholera, how often did you use ORS solution for the maintenance therapy? | | | | | 1 | 2 | | 3 | 4 | 5 | na |
|  | Over the past 12 months, when treating young children with diarrhoea, how often did you (or someone acting on your behalf) provide caretakers (e.g., mothers) with packets of ORS for home-use? | | | | | 1 | 2 | | 3 | 4 | 5 | na |

[FOR TB ONLY]

| **Never**  **1** | | **Rarely**  **2** | **Sometimes**  **3** | **Often**  **4** | **Very often**  **5** | | | **Not applicable**  **na** | | | | |
| --- | --- | --- | --- | --- | --- | --- | --- | --- | --- | --- | --- | --- |
|  | Over the past 12 months, when treating new active tuberculosis patients, how often did you prescribe a treatment regimen of 5 months or less? | | | | | 1 | 2 | | 3 | 4 | 5 | na |
|  | Over the past 12 months, when treating new active tuberculosis patients, how often did you (or someone acting on your behalf) ensure that the treatment was taken for at least 2 months in the presence of a health worker? | | | | | 1 | 2 | | 3 | 4 | 5 | na |
|  | Over the past 12 months, before initiating treatment with new active tuberculosis patients, how often did you (or someone acting on your behalf) provide health education on the importance of taking medication regularly? | | | | | 1 | 2 | | 3 | 4 | 5 | na |
|  | Over the past 12 months, when treating new active tuberculosis patients, how often did you (or someone acting on your behalf) notify the health authority about the new patients? | | | | | 1 | 2 | | 3 | 4 | 5 | na |
|  | Over the past 12 months, when treating HIV-infected individuals, how often did you recommend preventive chemotherapy for tuberculosis? | | | | | 1 | 2 | | 3 | 4 | 5 | na |

[FOR CONTRACEPTION ONLY]

| **Never**  **1** | | **Rarely**  **2** | **Sometimes**  **3** | **Often**  **4** | **Very often**  **5** | | | **Not applicable**  **na** | | | | |
| --- | --- | --- | --- | --- | --- | --- | --- | --- | --- | --- | --- | --- |
|  | Over the past 12 months, before providing intrauterine devices (IUDs), how often did you perform a pelvic/genital examination? | | | | | 1 | 2 | | 3 | 4 | 5 | na |
|  | Over the past 12 months, before providing combined oral contraceptives (COCs), how often did you perform a pelvic/genital examination? | | | | | 1 | 2 | | 3 | 4 | 5 | na |
|  | Over the past 12 months, when providing intrauterine devices (IUDs), how often did you recommend a follow-up visit after the first menses or 3-6 weeks following insertion? | | | | | 1 | 2 | | 3 | 4 | 5 | na |
|  | Over the past 12 months, when providing combined oral contraceptives (COCs), how often did you recommend a follow-up visit? | | | | | 1 | 2 | | 3 | 4 | 5 | na |
|  | Over the past 12 months, before providing combined oral contraceptives (COCs), how often did you screen for high blood pressure? | | | | | 1 | 2 | | 3 | 4 | 5 | na |

[FOR MALARIA ONLY]

| **Never**  **1** | | **Rarely**  **2** | **Sometimes**  **3** | **Often**  **4** | **Very often**  **5** | | | **Not applicable**  **na** | | | | |
| --- | --- | --- | --- | --- | --- | --- | --- | --- | --- | --- | --- | --- |
|  | Over the past 12 months, when treating young children, how often did you enquire about their and their caretakers' home-use of insecticide-treated nets? | | | | | 1 | 2 | | 3 | 4 | 5 | na |
|  | Over the past 12 months, when treating young children, how often did you recommend caretakers to use insecticide-treated nets for their young children? | | | | | 1 | 2 | | 3 | 4 | 5 | na |
|  | Over the past 12 months, when treating young children, how often did you inform caretakers who used insecticide-treated nets of the need to regularly re-treat their nets? | | | | | 1 | 2 | | 3 | 4 | 5 | na |
|  | Over the past 12 months, when treating young children and pregnant women, how often did you (or someone acting on your behalf) provide caretakers and pregnant women with an insecticide-treated nets for home-use? | | | | | 1 | 2 | | 3 | 4 | 5 | na |
|  | Over the past 12 months, when treating young children, how often did you inform caretakers that torn insecticide-treated nets are worse than no insecticide-treated nets? | | | | | 1 | 2 | | 3 | 4 | 5 | na |

[FOR ORT ONLY]

Please answer all questions by circling all of the correct answers.

1. Which one of the following methods is most effective in teaching caretakers (e.g., mothers) how to give Oral Rehydration Therapy (ORT)?

|  | |  |
| --- | --- | --- |
|  | The doctor explains how it is done | A |
|  | Posters on the clinic walls show how ORT is given | B |
|  | A nurse or health worker demonstrates ORT | C |
|  | The mother practises giving ORT with the guidance of a health worker | D |
|  | The mother is given an illustrated pamphlet that explains how ORT is carried out | E |

1. Which of the following might happen if Oral Rehydration Salts (ORS) solution was mixed with only half of the required amount of water and used to treat a child with diarrhoea and dehydration?

|  | | **Please circle all** |
| --- | --- | --- |
|  | The solution would be an "improved ORS" and cause the stool volume to be reduced and the duration of diarrhoea to be shortened | A |
|  | The child would develop hypernatraemia | B |
|  | The child would refuse to drink the solution | C |
|  | The child would develop paralytic ileus and abdominal distension | D |
|  | The child would become extremely thirsty | E |

1. Which of the following "home fluids" can be safely used to prevent dehydration in children with diarrhoea?

|  | | **Please circle all** |
| --- | --- | --- |
|  | Rice water | A |
|  | Cereal gruel with a small amount of salt added | B |
|  | Cola drink | C |
|  | Soup made from cooked legumes | D |
|  | Commercial fruit drink | E |

1. For which of the following situations is Oral Rehydration Therapy using Oral Rehydration Salts (ORS) solution not satisfactory?

|  | | **Please circle all** |
| --- | --- | --- |
|  | Maintenance therapy for an infant with diarrhoea | A |
|  | Rehydration of a child with non-severe dehydration due to cholera | B |
|  | Rehydration of a child with non-severe dehydration due to enterotoxigenic E. coli | C |
|  | Rehydration of a comatose child with severe dehydration and shock due to diarrhoea | D |
|  | Maintenance therapy of a child with cholera | E |

1. Which of the following measures are considered to be cost-effective with regard to the prevention of diarrhoea in young children?

|  | | **Please circle all** |
| --- | --- | --- |
|  | Control of flies | A |
|  | Hand-washing after defecation, before preparing food, and before eating | B |
|  | Exclusive breast-feeding for the first 4-6 months of life and continued breast-feeding for at least one year | C |
|  | Immunization for measles at 9 months of age | D |
|  | Providing caretakers of children with diarrhoea with packets of Oral Rehydration Salts (ORS) for home-use | E |

[FOR TB ONLY]

Please answer all questions by circling all of the correct answers.

1. What factors accelerate the progression from tuberculosis infection to disease:

|  | | **Please circle all** |
| --- | --- | --- |
|  | Malnutrition | A |
|  | HIV infection | B |
|  | Diabetes | C |
|  | Long-term treatment with corticosteroids or immunosuppressive medications | D |
|  | Bacillus Calmette-Guerin (BCG) vaccination | E |

1. All of the following are first line therapy for TB except:

|  | |  |
| --- | --- | --- |
|  | Isoniazid | A |
|  | Rifampin | B |
|  | Streptomycin | C |
|  | Cycloserine | D |
|  | Ethambutol | E |

1. The major side effect of isoniazid therapy is:

|  | |  |
| --- | --- | --- |
|  | Gastritis | A |
|  | Hepatitis | B |
|  | Diarrhoea | C |
|  | Tubular necrosis | D |
|  | Optic neuritis | E |

1. Which one of the following is the best indicator of a patient not being infectious?

|  | |  |
| --- | --- | --- |
|  | Patient has received at least 2 weeks of TB medications | A |
|  | Patient has no cough | B |
|  | Patient has three negative acid-fast bacilli (AFB) sputum smears on three consecutive days | C |

1. The minimum duration of therapy for culture-proven active TB is:

|  | |  |
| --- | --- | --- |
|  | 2 weeks | A |
|  | 6 weeks | B |
|  | 2 months | C |
|  | 6 months | D |
|  | 9 months | E |
|  | 12 months | F |

[FOR CONTRACEPTION ONLY]

Please indicate whether each of the following statements is True or False.

|  | | **True** | **False** |
| --- | --- | --- | --- |
|  | A woman can have a copper-bearing intrauterine device (IUD) inserted any time within the first 12 days after the start of menstrual bleeding, at her convenience, not just during menstruation. | True | False |
|  | Spotting or light bleeding between menstrual periods is common during the first 3–6 months of copper-bearing intrauterine device (IUD) use. It is not harmful and usually decreases over time. | True | False |
|  | Copper-bearing IUD should always be removed if the intrauterine device (IUD) user is diagnosed with pelvic inflammatory disease (PID). | True | False |
|  | One follow-up visit after the first menses or 3–6 weeks following copper-bearing intrauterine device (IUD) insertion is sufficient. | True | False |
|  | The most commonly used IUD, the CuT380a, is approved for ten years of use after insertion. | True | False |

[FOR MALARIA ONLY]

Please indicate whether each of the following statements is True or False.

|  | | **True** | **False** |
| --- | --- | --- | --- |
|  | Insecticide-treated nets that are torn are no longer effective and should not be used. | True | False |
|  | The use of insecticide-treated nets can reduce the number of bites in sleepers without nets in the same houses. | True | False |
|  | The use of untreated nets can divert extra biting to sleepers without nets in the same houses. | True | False |
|  | Insecticide-treated nets need regular re-treatment to remain effective while long-lasting insecticidal nets remain effective for a long time and after many washes, without the need for re-treatment. | True | False |
|  | Insecticide-treated nets ability to reduce the number of malaria episodes in communities with stable malaria has *not* been demonstrated. | True | False |

1. Please indicate how important the following issues are for you to improve your work?

| **Unimportant**  **1** | | Somewhat important  **2** | **Moderately important**  **3** | **Important**  **4** | **Very Important**  **5** | | | | | |
| --- | --- | --- | --- | --- | --- | --- | --- | --- | --- | --- |
|  | Financial incentives (e.g., better pay) | | | | | 1 | 2 | 3 | 4 | 5 |
|  | More staff | | | | | 1 | 2 | 3 | 4 | 5 |
|  | More training | | | | | 1 | 2 | 3 | 4 | 5 |
|  | More feedback on staff performance | | | | | 1 | 2 | 3 | 4 | 5 |
|  | More/better equipment or supplies | | | | | 1 | 2 | 3 | 4 | 5 |
|  | Better security | | | | | 1 | 2 | 3 | 4 | 5 |
|  | Better physical environment | | | | | 1 | 2 | 3 | 4 | 5 |
|  | Higher quality of available research | | | | | 1 | 2 | 3 | 4 | 5 |
|  | More access to peers / networks | | | | | 1 | 2 | 3 | 4 | 5 |
|  | More locally applicable research | | | | | 1 | 2 | 3 | 4 | 5 |
|  | Other (please specify): ____________________ | | | | | 1 | 2 | 3 | 4 | 5 |

1. Please indicate whether you were involved in any the following activities (either formally or informally) to improve your clinical practice and/or the quality of your working life over the past 12 months?

|  | | **Yes**  **1** | **No**  **2** | **Not sure**  **0** |
| --- | --- | --- | --- | --- |
|  | Working with researchers or researcher groups | ❑ | ❑ | ❑ |
|  | Working with patient groups | ❑ | ❑ | ❑ |
|  | Working with representatives of non-governmental organizations (NGOs) | ❑ | ❑ | ❑ |
|  | Working with representatives of for-profit organizations (e.g., pharmaceutical companies) | ❑ | ❑ | ❑ |
|  | Working with policy-makers | ❑ | ❑ | ❑ |
|  | Working with peers to exchange ideas, experiences and best practices | ❑ | ❑ | ❑ |

1. Please indicate to what extent are scientific journals from the following places likely to influence your clinical practice.

| **Very unlikely**  **1** | | **Unlikely**  **2** | **Neutral**  **3** | **Likely**  **4** | **Very likely**  **5** | | | | | |
| --- | --- | --- | --- | --- | --- | --- | --- | --- | --- | --- |
|  | High-income countries | | | | | 1 | 2 | 3 | 4 | 5 |
|  | Your region (INSERT REGION NAME) | | | | | 1 | 2 | 3 | 4 | 5 |
|  | Your country of practice | | | | | 1 | 2 | 3 | 4 | 5 |

1. Please indicate to what extent is research performed in the following places likely to influence your clinical practice.

| **Very unlikely**  **1** | | **Unlikely**  **2** | **Neutral**  **3** | **Likely**  **4** | **Very likely**  **5** | | | | | |
| --- | --- | --- | --- | --- | --- | --- | --- | --- | --- | --- |
|  | High-income countries | | | | | 1 | 2 | 3 | 4 | 5 |
|  | Your region (INSERT REGION NAME) | | | | | 1 | 2 | 3 | 4 | 5 |
|  | Your country of practice | | | | | 1 | 2 | 3 | 4 | 5 |

1. Please indicate how you rate the quality of the research performed in the following places.

| **Extremely poor**  **1** | | **Below average**  **2** | **Average**  **3** | **Above average**  **4** | **Excellent**  **5** | | | | | |
| --- | --- | --- | --- | --- | --- | --- | --- | --- | --- | --- |
|  | High-income countries | | | | | 1 | 2 | 3 | 4 | 5 |
|  | Your region (INSERT REGION NAME) | | | | | 1 | 2 | 3 | 4 | 5 |
|  | Your country of practice | | | | | 1 | 2 | 3 | 4 | 5 |

1. Please indicate how you rate the applicability of the research performed in the following places to your local settings?

| **Extremely poor**  **1** | | **Below average**  **2** | **Average**  **3** | **Above average**  **4** | **Excellent**  **5** | | | | | |
| --- | --- | --- | --- | --- | --- | --- | --- | --- | --- | --- |
|  | High-income countries | | | | | 1 | 2 | 3 | 4 | 5 |
|  | Your region (INSERT REGION NAME) | | | | | 1 | 2 | 3 | 4 | 5 |
|  | Your country of practice | | | | | 1 | 2 | 3 | 4 | 5 |

Finally, we would like to ask you a few questions about your practice and yourself.

1. What is your year of birth? __________
2. What is your gender? 1. Male ❑ 2. Female ❑
3. Please indicate whether you practice primarily as a: [RESPONSE CATEGORIES WILL DEPEND ON EACH COUNTRY'S SAMPLE]

|  | |  |
| --- | --- | --- |
|  | Primary care physician/General practitioner | ❑ |
|  | Specialist physician | ❑ |
|  | Nurse | ❑ |
|  | Health worker | ❑ |
|  | Other (please specify): __________________________ | ❑ |

1. Please estimate the percentage of your time that you spend doing each of the following:

|  | | **%** |
| --- | --- | --- |
|  | Clinical practice |  |
|  | Research |  |
|  | Teaching |  |
|  | Administration |  |
|  | Other (please specify): ___________________ |  |
|  |  | **Total = 100** |

1. Please indicate if you have completed the following degrees. If so, when?

|  | | **Yes**  **If so, when** | **Specialty, if any If so, when** | **No** |
| --- | --- | --- | --- | --- |
|  | Medical degree (MD, MBBCh) | ❑ year: _____ | ❑ year: _____ | ❑ |
|  | Nursing degree (RN) | ❑ year: _____ | ❑ year: _____ | ❑ |
|  | Master's degree (e.g., MPH, MSc) | ❑ year: _____ |  | ❑ |
|  | Doctorate degree (e.g., PhD, DPhil, DSc, ScD) | ❑ year: _____ |  | ❑ |

1. Please indicate how well you read and write English? Please note the following definitions: “very well” means you can read/write scientific reports and articles and give lectures or presentations (i.e., as good or almost as good as mother tongue); “well” means you can read newspaper articles/contribute to writing scientific papers/contribute to discussions (i.e., advanced); and “a little” means you can read signs, write short notes, ask for directions, or not at all.

|  | |  |
| --- | --- | --- |
|  | Very well / almost as good as mother tongue | ❑ |
|  | Well / advanced | ❑ |
|  | A little / not at all | ❑ |

[FOR ORT ONLY]

Approximately how many infants and young children with diarrhoea have you seen over the past 12 months?

_______________ (number of cases encountered)

[FOR TB ONLY]

Approximately how many patients with latent tuberculosis infection have you seen over the past 12 months?

_______________ (number of cases encountered)

[FOR CONTRACEPTION ONLY]

Approximately how many women and/or couples have you counseled about family planning over the past 12 months?

_______________ (number of cases encountered)

[FOR MALARIA ONLY]

1. Approximately how many children with malaria-related fevers have you seen over the past 12 months?

_______________ (number of cases encountered)

1. Approximately how many pregnant women have you seen over the past 12 months?

_______________ (number of cases encountered)

1. Are you aware of any recommendations or guidelines concerning [INSERT HEALTH TOPIC]? If yes, what is the source?

1. Yes ❑ Source: ___________________________________________

___________________________________________

2. No ❑

1. Would you characterize the operating authority of your facility(ies) as government, non-governmental organization or for-profit? Please tick *all* that apply if you work in more than one facility.

1. Government ❑ 2. Non-governmental organization ❑ 3. For-profit ❑

1. Would you characterize the service area of your facility(ies) as rural, urban or mixed? Please tick *all* that apply if you work in more than one facility.

1. Rural ❑ 2. Urban ❑ 3. Mixed ❑

1. How would you define the organization of your practice(s)? Please tick *all* that apply if you work in more than one facility. [RESPONSE CATEGORIES WILL DEPEND ON EACH COUNTRY'S SAMPLE]

|  | |  |
| --- | --- | --- |
|  | Solo/individual practice | ❑ |
|  | Group practice | ❑ |
|  | Hospital | ❑ |
|  | Community health centre | ❑ |
|  | Other (please specify): ___________________ | ❑ |

1. Does your facility (Do your facilities) have the following items available today?

|  | | **Yes**  **1** | **No**  **2** |
| --- | --- | --- | --- |
|  | Supplies to mix Oral Rehydration Salts (ORS), cups and spoons (FOR ORT ONLY) | ❑ | ❑ |
|  | Posters on the clinic walls to show how Oral Rehydration Therapy (ORT) is given? (FOR ORT ONLY) | ❑ | ❑ |
|  | Illustrated pamphlet that explains how Oral Rehydration Therapy (ORT) is given out (FOR ORT ONLY) | ❑ | ❑ |
|  | Oral Rehydration Salts (ORS) (FOR ORT ONLY) | ❑ | ❑ |
|  | Recommended intravenous (IV) fluid for severe dehydration (FOR ORT ONLY) | ❑ | ❑ |
|  | Insecticide-treated nets (ITNs) [FOR MALARIA ONLY] | ❑ | ❑ |
|  | Anti-tuberculosis drugs [FOR TB ONLY] | ❑ | ❑ |
|  | Intrauterine devices (IUDs) [FOR CONTRACEPTION ONLY] | ❑ | ❑ |

1. Please summarize in point form what you think is the most salient findings from the body of all available research on [INSERT HEALTH TOPIC].

**___________________________________________________________________________________________ ______________________________________________________________________________________________________________________________________________________________________________________**

**___________________________________________________________________________________________**

**Source (if available): _______________________________________________________________________**

1. Would you be willing to let us contact you again if other questions emerge from this research?

1. Yes ❑ 2. No ❑

**Thank you for completing the questionnaire.**

Please return the completed questionnaire in the large envelope provided.

As a small token of our appreciation, we are pleased to provide you with a selection of WHO publications that we hope will be of use to both you and your patients. To receive these publications, simply complete the attached request form, place it in the small envelope addressed to Emmanuel Guindon of the World Health Organization, and seal the small envelope. Please insert this sealed envelope along with your completed questionnaire in the large return envelope addressed to the local investigator leading this study and seal the large envelope. Please be assured that your request form will be kept separate from your completed questionnaire.

ID #: __________

(Your responses will be kept confidential and data will not be reported in ways that could potentially identify you or your facility.)

**Additional Thoughts (Optional)**

Do you have any comments regarding any of the questions?

(If the space provided is insufficient to accommodate all your ideas, please feel free to attach additional pages.)

___________________________________________________________________________________________ ______________________________________________________________________________________________________________________________________________________________________________________

___________________________________________________________________________________________

______________________________________________________________________________________________________________________________________________________________________________________

___________________________________________________________________________________________

______________________________________________________________________________________________________________________________________________________________________________________

___________________________________________________________________________________________

______________________________________________________________________________________________________________________________________________________________________________________

___________________________________________________________________________________________

______________________________________________________________________________________________________________________________________________________________________________________

___________________________________________________________________________________________

______________________________________________________________________________________________________________________________________________________________________________________

___________________________________________________________________________________________

______________________________________________________________________________________________________________________________________________________________________________________

___________________________________________________________________________________________

______________________________________________________________________________________________________________________________________________________________________________________

___________________________________________________________________________________________

______________________________________________________________________________________________________________________________________________________________________________________

___________________________________________________________________________________________
