# Supplementary material for: Bridging the gaps among research, policy and practice in ten low- and middle-income countries: Development and testing of a questionnaire for health-care providers
Source: Health Res Policy Syst. 2010 Jan 29;8:3. doi: 10.1186/1478-4505-8-3 (PMC2825186; doi:10.1186/1478-4505-8-3)
Supplement: Additional file 2 — Sample. Table S1: Sample sizes and response rates by country and health topics. Table S2: Sampling design by country and health topics. [file 1478-4505-8-3-S2.DOC]

**Additional file 2: Sample**

**China**

*Family planning*. A stratified random sampling process was used to sample 120 health-care providers (primarily specialists) who provided care to women seeking contraception. The sampling frame was constructed from an existing list of family planning centres. The sample was stratified by geographic location (Sichuan province in the south-west and the Liaoning province in the north-east) and by types of facilities (Maternal and Child Health (MCHs) clinics, Department of Obstetrics & Gynecology in hospitals and Family Planning service institutions). 115 questionnaires were collected yielding a 0.96 response rate.

*Tuberculosis*. A cluster random sampling process was used to sample 120 health-care providers (primarily primary care physicians) who provided care to patients with tuberculosis. The sampling frame was constructed from an existing list of TB control centres in the Hebei Province which surrounds Beijing. First, one county (Weichang) was randomly selected. Secondly, 97 facilities from Weichang county, stratified by administrative authorities (county, township and village), were randomly selected. 116 questionnaires were collected yielding a 0.97 response rate.

**Ghana**

*Oral Rehydration Therapy (ORT).* A stratified cluster random sampling process was used to sample 140 health-care providers (primarily primary care physicians and nurses) that provided care to children with diarrhoea. The sampling frame was constructed from an existing lists of government health facilities (from the Ministry of Health) and private health facilities (from the Society of Private Medical and Dental Practitioners). 48 facilities, stratified by geographic location (Greater Accra Region and Ashanti Region) and types of providers (public vs. private) were randomly selected. 110 questionnaires were collected, of which 12 were excluded by the country investigators because of serious incompleteness, leaving 98 for analysis, yielding a 0.70 response rate.

*Malaria.* The same sampling strategy was used to sample 140 health-care providers (primarily primary care physicians and nurses) that were involved in the prevention of malaria. 122 questionnaires were collected, of which 15 were excluded by the country investigators because of serious incompleteness, leaving 107 for analysis, yielding a 0.76 response rate.

**India**

*Tuberculosis.* A stratified cluster random sampling process was used to sample 100 Medical Officers in charge of Health Post (almost all primary care physicians) in the poor areas of Mumbai. The sampling frame was constructed from an existing list of health posts set up by the Municipal Corporation of Greater Mumbai Health department. The list was compiled by ALERT India, an NGO working in Mumbai. 100 Health Posts (i.e. facilities), stratified by the number of Health Posts (≤ 6, 7-9, or ≥ 10) per Ward (municipal location area) were randomly selected. 100 questionnaires were collected yielding a 1.00 response rate.

*Oral Rehydration Therapy (ORT).* A stratified cluster random sampling process was used to sample 100 Medical Officers in charge of Dispensaries (almost all primary care physicians) in the poor areas of Mumbai. The list was compiled by ALERT India, an NGO working in Mumbai. The sampling frame was constructed from an existing list of Dispensaries set up by the Municipal Corporation of Greater Mumbai Health department. 100 Dispensaries (i.e. facilities), stratified by the number of Dispensaries (≤ 6, 7-9, or ≥ 10) per Ward (municipal location area) were randomly selected. 100 questionnaires were collected yielding a 1.00 response rate.

**Iran**

*Tuberculosis.* A stratified cluster random sampling process was used to sample 128 primary care physicians and specialists providing care to patients with tuberculosis. The sampling frame was constructed from an existing human resources database from each province's medical sciences university. First, 11 Health Districts were randomly selected. Secondly, 128 facilities, stratified by geographic location (Systan and Baluchetan province, Golestan province and Hormozgan province) and types of providers (government GPs, private GPs and specialists) were randomly selected. 108 questionnaires were collected yielding a 0.84 response rate.

**Kazakhstan**

*Family planning*. The total population (110) of gynecologists working at the primary care level in the area of family planning that provided care to women seeking contraception in Almaty city were sampled. The sampling frame was constructed from an existing database from Department of Health of Almaty. 110 questionnaires were collected yielding a 1.00 response rate.

**Laos**

*Family planning*. A stratified random sampling process was used to sample 106 health-care providers (primarily primary care physicians) providing care to women seeking contraception in the capital city Vientiane and the provinces of Vientiane, Borikhamsay and Savannakheth. The sampling frame was constructed from existing lists of health care providers obtained from the Department of Human Resources for Health, Ministry of Health and four provincial health departments. The sample was stratified by facility types (central, provincial, and district hospitals). 105 questionnaires were collected yielding a 0.99 response rate.

*Malaria*. The same sampling strategy was used to sample 136 health-care providers (primarily primary care physicians) involved in the prevention of malaria. 136 questionnaires were collected yielding a 1.00 response rate.

**Mexico**

*Family planning*. A simple random sampling process was used to sample 122 health-care providers in Mexico City and the states of Mexico, Nuevo Leon and Jalisco. The sampling frame was constructed from five distinct sources (physicians working for the Mexican Foundation for Family Planning (MEXFAM), physicians working for the Instituto de Seguridad y Servicios Sociales de los Trabajadores del Estado (ISSSTE) (Federal Employees Social Services Institution), physicians working for the Secretaría de Salud (SSa) (Ministry of Health) in five facilities of Mexico city, physicians involved in family planning attending a training course at the Desarrollo Integral de la Familia (DIF) (Family Development) and at the Instituto Mexicano del Seguro Social (IMSS) (Mexican Institute for Social Security) and, private physicians involved in family planning attending a training course). 108 questionnaires were collected yielding a 0.89 response rate.

*Tuberculosis*. A simple random sampling process was used to sample 123 health-care providers providing care to patients with tuberculosis in Mexico City and the states of Mexico, Nuevo Leon and Jalisco. The sampling frame was constructed from five distinct sources (physicians working for the Instituto Nacional de Enfermedades Respiratorias (INER) (National Institute of Respiratory Diseases), physicians working for the Instituto de Seguridad y Servicios Sociales de los Trabajadores del Estado (ISSSTE), physicians working for the Secretaría de Salud (SSA) in five facilities of Mexico city, physicians attending a training course at the Instituto Mexicano del Seguro Social (IMSS) and, private physicians involved in tuberculosis care attending a training course). 60 questionnaires were collected yielding a 0.49 response rate.

**Pakistan**

*Oral Rehydration Therapy (ORT).* A simple random sampling process was used to sample 105 health-care providers (primarily primary care physicians) providing care to children with diarrhoea in the North West Frontier Province. The sampling frame was constructed from an existing list of health-care providers from the Department of Health. 63 questionnaires were collected yielding a 0.60 response rate.

**Senegal**

*Malaria*. A simple random sampling process was used to sample 100 health-care providers (primary care physicians, nurses and health-workers) involved in the prevention of malaria in Dakar (the city of Dakar and the departments of Pikine and Guédiéwaye) and Thiès (city of Thiès, and rural communities of Mont-Roland, Pout, Kayar and Diamniadio). The sampling frame was created from lists of providers at 8 institutions (selected based on geographical location) and a list of researchers who were also health-care providers created for phase I of this project. 51 questionnaires were collected yielding a 0.55 response rate.

*Oral Rehydration Therapy (ORT).* The same sampling strategy was used to sample 100 health-care providers (primarily nurses) that provided care to children with diarrhoea. 44 questionnaires were collected yielding a 0.44 response rate.

**Tanzania**

*Malaria*. A purposive sampling process was used to sample 121 regional or district medical officers (primarily primary care physicians) who were attending a meeting on malaria and IMCI. 75 questionnaires were collected yielding a 0.62 response rate.

**Table S1: Sample sizes and response rates by country and health topics**

|  | Malaria | Family Planning | ORT | Tuberculosis | Total |
| --- | --- | --- | --- | --- | --- |
| China | - | 115/120 | - | 116/120 | 231/240 |
| Ghana | 107/140 | - | 98/140 | - | 205/280 |
| India | - | - | 100/100 | 100/100 | 200/200 |
| Iran | - | - | - | 108/128 | 108/128 |
| Kazakhstan | - | 110/110 | - | - | 110/110 |
| Laos | 136/136 | 105/106 | - | - |  |
| Mexico | - | 108/122 |  | 60/123 | 168/245 |
| Pakistan |  | - | 63/105 | - | 63/105 |
| Senegal | 51/100 | - | 44/100 | - | 95/200 |
| Tanzania | 78/121 | - | - | - | 78/121 |
| Total | 372/497 | 438/458 | 305/ 445 | 384/471 | 1,499/1,629 |

**Table S2: Sampling design by country and health topics**

| Country/Topics | Sampling process | Unit of randomization | Stratification | Geographic area |
| --- | --- | --- | --- | --- |
| *China* |  |  |  |  |
| - Family Planning | Stratified Random | Providers | Geographic location  Type of facilities | Sichuan province  Liaoning province |
| - Tuberculosis | Cluster Random (county, facilities) | Facilities | n/a | Weichang county |
| *Ghana* |  |  |  |  |
| - Malaria - ORT | Stratified Cluster Random (facilities) | Facilities | Geographic location | Greater Accra  Ashanti Region |
| *India* |  |  |  |  |
| - ORT - Tuberculosis | Stratified Cluster Random (facilities) | Facilities | Number of Health Posts/Dispensaries per Ward | Poor areas of Mumbai |
| *Iran* |  |  |  |  |
| - Tuberculosis | Stratified Cluster Random (health districts, facilities) | Facilities | Geographic location | Systan and Baluchetan province  Golestan province Hormozgan province |
| *Kazakhstan* |  |  |  |  |
| - Family Planning | Whole population (gynecologists) | n/a | n/a | Almaty city |
| *Laos* |  |  |  |  |
| - Malaria - Family Planning | Stratified Cluster Random | Providers | Facilities | Vientiane city  Vientiane province  Borikhamsay province Savannakheth province |
| *Mexico* |  |  |  |  |
| - Family Planning - Tuberculosis | Simple random | Providers | n/a | Mexico City  States of Mexico, Nuevo Leon and Jalisco |
| *Pakistan* |  |  |  |  |
| - ORT | Simple random | Providers | n/a | North West Frontier Province |
| *Senegal* |  |  |  |  |
| - Malaria - ORT | Simple random | Providers | n/a | Dakar  Thiès |
| *Tanzania* |  |  |  |  |
| - Malaria | Purposive | n/a | n/a | n/a |

Note: ORT: Oral rehydration therapy; n/a: not applicable
