# Supplementary material for: Bridging the gaps among research, policy and practice in ten low- and middle-income countries: Development and testing of a questionnaire for health-care providers
Source: Health Res Policy Syst. 2010 Jan 29;8:3. doi: 10.1186/1478-4505-8-3 (PMC2825186; doi:10.1186/1478-4505-8-3)
Supplement: Additional file 3 — Factor analyses. Additional file 3.1: Factor analyses: Awareness, access and use of electronic/online sources of information. Table S3.1-1, Table S3.1-2 and Table S3.1-3. Additional file 3.2: Factor analyses: Awareness, access and use of paper sources of information. Table S3.2-1, Table S3.2-2: and Table S3.2-3. Additional file 3.3: Factor analyses: Trust in types of sources of information. Table S3.3-1, Table S3.3-2 and Table S3.3-3. Additional file 3.4: Factor analyses: Extent of research utilization. Table S3.4-1. Additional file 3.5: Factor analyses: Attitudes to issues needed to be addressed to improve practice. Table S3.5-1, Table S3.5-2, Table S3.5-3, Table S3.5-4 and Table S3.5-5. [file 1478-4505-8-3-S3.DOC]

**Additional file 3: Factor analyses**

**Additional file 3.1: Factor analyses: Awareness, access and use of electronic/online sources of information**

Bartlett test of sphericity: Chi-square = 3942.924; d.f. = 91; p-value = 0.000

Kaiser-Meyer-Olkin Measure of Sampling Adequacy: KMO = 0.904

**Table S3.1-1**

**Factor analysis: Awareness, access and use of electronic/online sources of information**

--------------------------------------------------------------------------

Factor | Eigenvalue Difference Proportion Cumulative

-------------+------------------------------------------------------------

Factor1 | 6.15941 4.78540 0.6292 0.6292

Factor2 | 1.37401 0.70121 0.1404 0.7696

Factor3 | 0.67280 0.14605 0.0687 0.8383

Factor4 | 0.52675 0.14028 0.0538 0.8921

Factor5 | 0.38647 0.15023 0.0395 0.9316

Factor6 | 0.23624 0.04194 0.0241 0.9557

Factor7 | 0.19430 0.07247 0.0198 0.9756

Factor8 | 0.12183 0.06141 0.0124 0.9880

Factor9 | 0.06042 0.02960 0.0062 0.9942

Factor10 | 0.03082 0.01673 0.0031 0.9973

Factor11 | 0.01409 0.00546 0.0014 0.9988

Factor12 | 0.00863 0.00508 0.0009 0.9997

Factor13 | 0.00356 0.00386 0.0004 1.0000

Factor14 | -0.00030 . -0.0000 1.0000

--------------------------------------------------------------------------

**Table S3.1-2**

**Factor loading matrix –rotated (orthogonal varimax): Awareness, access and use of electronic/online sources of information**

-----------------------------------------------------------------

Variable | Factor1 Factor2 | Uniqueness

-------------+--------------------+------------------------------

Textbooks | 0.2302 0.5279 | 0.6683

Guidelines | 0.2637 0.5891 | 0.5834

Dare | 0.6463 | 0.5388

Cochrane Library | 0.7460 | 0.4147

HINARI | 0.6666 | 0.5260

Other open access | 0.6015 0.3295 | 0.5296

Int’l biblio | 0.6638 0.3823 | 0.4132

Regional biblio. | 0.6790 | 0.5015

Journals (high-income) | 0.6247 0.4308 | 0.4241

Journals (own region) | 0.5031 0.4903 | 0.5064

Journals (own country) | 0.2717 0.5642 | 0.6079

Reports (not-for-profit) | 0.7995 | 0.3128

Reports (for profit) | 0.7263 | 0.4411

Summaries (not-for-profit) | 0.2246 0.7770 | 0.3459

-----------------------------------------------------------------

(blanks represent abs(loading)<.222)

**Table S3.1-3**

**Factor loading matrix –rotated (oblique promax): Awareness, access and use of electronic/online sources of information**

-----------------------------------------------------------------

Variable | Factor1 Factor2 | Uniqueness

-------------+--------------------+------------------------------

Textbooks | 0.5285 | 0.6683

Guidelines | 0.5873 | 0.5834

Dare | 0.6772 | 0.5388

Cochrane Library | 0.8076 | 0.4147

HINARI | 0.7142 | 0.5260

Other open access | 0.5812 | 0.5296

Int’l biblio. | 0.6346 | 0.4132

Regional biblio. | 0.7207 | 0.5015

Journals (high-income) | 0.5714 0.2697 | 0.4241

Journals (own region) | 0.4083 0.3838 | 0.5064

Journals (own country) | 0.5553 | 0.6079

Reports (not-for-profit) | 0.8485 | 0.3128

Reports (for-profit) | 0.7787 | 0.4411

Summaries (not-for-profit) | 0.8202 | 0.3459

----------------------------------------------------------------

(blanks represent abs(loading)<.222)

**Additional file 3.2: Factor analyses: Awareness, access and use of paper sources of information**

Bartlett test of sphericity: Chi-square = 3541.584; d.f. = 28; p-value = 0.000

Kaiser-Meyer-Olkin Measure of Sampling Adequacy: KMO = 0.782

**Table S3.2-1**

**Factor analysis: Awareness, access and use of paper sources of information**

--------------------------------------------------------------------------

Factor | Eigenvalue Difference Proportion Cumulative

-------------+------------------------------------------------------------

Factor1 | 3.26451 2.48372 0.6265 0.6265

Factor2 | 0.78078 0.11638 0.1498 0.7764

Factor3 | 0.66441 0.36091 0.1275 0.9039

Factor4 | 0.30350 0.15278 0.0582 0.9621

Factor5 | 0.15071 0.11196 0.0289 0.9911

Factor6 | 0.03876 0.03074 0.0074 0.9985

Factor7 | 0.00802 0.00824 0.0015 1.0000

Factor8 | -0.00022 . -0.0000 1.0000

--------------------------------------------------------------------------

**Table S3.2-2**

**Factor loading matrix –rotated (orthogonal varimax): Awareness, access and use of paper sources of information**

----------------------------------------------------------------

Variable | Factor1 Factor2 | Uniqueness

-------------+--------------------+-----------------------------

Textbooks | 0.6313 | 0.5921

Guidelines | 0.2378 0.7415 | 0.3937

Journals (high-income) | 0.3918 0.4182 | 0.6716

Journals (own region) | 0.5013 0.3996 | 0.5890

Journals (own country) | 0.3817 0.4926 | 0.6117

Reports (not-for-profit) | 0.7147 0.2325 | 0.4351

Reports (for-profit) | 0.5954 0.2416 | 0.5871

Summaries (not-for-profit) | 0.7784 | 0.3793

----------------------------------------------------------------

(blanks represent abs(loading)<.143)

**Table S3.2-3**

**Factor loading matrix –rotated (oblique promax): Awareness, access and use of paper sources of information**

----------------------------------------------------------------

Variable | Factor1 Factor2 | Uniqueness

-------------+--------------------+-----------------------------

Textbooks | 0.6781 | 0.5921

Guidelines | 0.7571 | 0.3937

Journals (high-income) | 0.3128 0.3456 | 0.6716

Journals (own region) | 0.4416 0.2901 | 0.5890

Journals (own country) | 0.2786 0.4322 | 0.6117

Reports (not-for-profit) | 0.7330 | 0.4351

Reports (for-profit) | 0.5959 | 0.5871

Summaries (not-for-profit) | 0.8385 | 0.3793

----------------------------------------------------------------

(blanks represent abs(loading)<.143)

**Additional file 3.3: Factor analyses:** **Trust in types of sources of information**

Bartlett test of sphericity: Chi-square = 4162.774; d.f. = 28; p-value = 0.000

Kaiser-Meyer-Olkin Measure of Sampling Adequacy: KMO = 0.801

**Table S3.3-1**

**Factor analysis: Trust in types of sources of information**

--------------------------------------------------------------------------

Factor | Eigenvalue Difference Proportion Cumulative

-------------+------------------------------------------------------------

Factor1 | 3.38405 2.28229 0.6492 0.6492

Factor2 | 1.10176 0.74590 0.2114 0.8605

Factor3 | 0.35586 0.15996 0.0683 0.9288

Factor4 | 0.19589 0.06567 0.0376 0.9664

Factor5 | 0.13023 0.09666 0.0250 0.9914

Factor6 | 0.03357 0.02182 0.0064 0.9978

Factor7 | 0.01174 0.01198 0.0023 1.0000

Factor8 | -0.00023 . -0.0000 1.0000

--------------------------------------------------------------------------

**Table S3.3-2**

**Factor loading matrix –rotated (orthogonal varimax): Trust in types of sources of information**

----------------------------------------------------------------

Variable | Factor1 Factor2 | Uniqueness

-------------+--------------------+-----------------------------

Cohort study | 0.6115 | 0.6160

Systematic review of RCTs | 0.7605 0.1967 | 0.3830

Experience | 0.5542 | 0.6754

Case control | 0.5593 0.3723 | 0.5486

RCT | 0.8447 0.2160 | 0.2398

Case series | 0.3335 0.6118 | 0.5145

Expert opinion | 0.1779 0.8059 | 0.3188

Case report | 0.1496 0.7339 | 0.4390

----------------------------------------------------------------

(blanks represent abs(loading)<.142)

**Table S3.3-3**

**Factor loading matrix –rotated (oblique promax): Trust in types of sources of information**

----------------------------------------------------------------

Variable | Factor1 Factor2 | Uniqueness

-------------+--------------------+-----------------------------

Cohort study | 0.6468 | 0.6160

Systematic review of RCTs | 0.7846 | 0.3830

Experience | 0.5735 | 0.6754

Case control | 0.5139 0.2523 | 0.5486

RCT | 0.8722 | 0.2398

Case series | 0.1981 0.5801 | 0.5145

Expert opinion | 0.8381 | 0.3188

Case report | 0.7667 | 0.4390

----------------------------------------------------------------

(blanks represent abs(loading)<.142)

**Additional file S3.4: Factor analyses: Extent of research utilization**

Bartlett test of sphericity: Chi-square = 6734.005; d.f. = 15; p-value = 0.000

Kaiser-Meyer-Olkin Measure of Sampling Adequacy: KMO = 0.882

**Table S3.4-1**

**Factor analysis: Extent of research utilization**

--------------------------------------------------------------------------

Factor | Eigenvalue Difference Proportion Cumulative

-------------+------------------------------------------------------------

Factor1 | 4.23723 3.87931 0.8844 0.8844

Factor2 | 0.35791 0.18308 0.0747 0.9591

Factor3 | 0.17483 0.15674 0.0365 0.9956

Factor4 | 0.01809 0.01484 0.0038 0.9994

Factor5 | 0.00325 0.00348 0.0007 1.0000

Factor6 | -0.00023 . -0.0000 1.0000

--------------------------------------------------------------------------

**Additional File S3.5: Factor analyses: Attitudes to issues needed to be addressed to improve practice**

Bartlett test of sphericity: Chi-square = 4394.440; d.f. = 45; p-value = 0.000

Kaiser-Meyer-Olkin Measure of Sampling Adequacy: KMO = 0.868

**Table S3.5-1**

**Factor analysis: Attitudes to issues needed to be addressed to improve practice**

--------------------------------------------------------------------------

Factor | Eigenvalue Difference Proportion Cumulative

-------------+------------------------------------------------------------

Factor1 | 3.72113 2.89580 0.6642 0.6642

Factor2 | 0.82533 0.27086 0.1473 0.8115

Factor3 | 0.55447 0.35039 0.0990 0.9105

Factor4 | 0.20408 0.04896 0.0364 0.9469

Factor5 | 0.15512 0.07762 0.0277 0.9746

Factor6 | 0.07750 0.03479 0.0138 0.9884

Factor7 | 0.04271 0.02729 0.0076 0.9960

Factor8 | 0.01542 0.00840 0.0028 0.9988

Factor9 | 0.00701 0.00727 0.0013 1.0000

Factor10 | -0.00026 . -0.0000 1.0000

--------------------------------------------------------------------------

**Table S3.5-2**

**Factor loading matrix –rotated (orthogonal varimax), 3-factors retained: Attitudes to issues needed to be addressed to improve practice**

---------------------------------------------------------------------------

Variable | Factor1 Factor2 Factor3 | Uniqueness

-------------+------------------------------+------------------------------

Financial incentives | 0.4501 0.2234 | 0.7451

More staff | 0.5885 0.1448 | 0.6297

Training | 0.5840 0.3161 0.1391 | 0.5397

Feedback on performance | 0.6778 0.3046 | 0.4428

Equipment/supplies | 0.5273 0.2878 0.2507 | 0.5763

Security | 0.1859 0.2663 0.7210 | 0.3747

Physical environment | 0.2949 0.2148 0.5931 | 0.5151

Higher quality of research | 0.1869 0.6389 0.3429 | 0.4393

Access to peers/networks | 0.1668 0.7385 0.2507 | 0.3640

Locally applicable research | 0.2309 0.7063 | 0.4385

---------------------------------------------------------------------------

(blanks represent abs(loading)<.138)

**Table S3.5-3**

**Factor loading matrix –rotated (orthogonal varimax), 2-factors retained: Attitudes to issues needed to be addressed to improve practice**

-----------------------------------------------------------------

Variable | Factor1 Factor2 | Uniqueness

-------------+--------------------+------------------------------

Financial incentives | 0.4903 | 0.7447

More staff | 0.6089 | 0.6210

Training | 0.3313 0.5730 | 0.5619

Feedback on performance | 0.2949 0.6289 | 0.5174

Equipment/supplies | 0.3527 0.5541 | 0.5686

Security | 0.4907 0.3165 | 0.6591

Physical environment | 0.4249 0.3854 | 0.6710

Higher quality of research | 0.7396 0.2065 | 0.4103

Access to peers/networks | 0.7727 0.1700 | 0.3741

Locally applicable research | 0.6409 0.2153 | 0.5429

-----------------------------------------------------------------

(blanks represent abs(loading)<.138)

**Table S3.5-4**

**Factor loading matrix –rotated (oblique promax), 3-factors retained: Attitudes to issues needed to be addressed to improve practice**

--------------------------------------------------------------------------

Variable | Factor1 Factor2 Factor3 | Uniqueness

-------------+------------------------------+-----------------------------

Financial incentives | 0.4648 0.1761 | 0.7451

More staff | 0.6406 | 0.6297

Training | 0.1894 0.5650 | 0.5397

Feedback on performance | 0.1688 0.6917 | 0.4428

Equipment/supplies | 0.1404 0.4835 | 0.5763

Security | 0.7492 | 0.3747

Physical environment | 0.1618 0.5942 | 0.5151

Higher quality of research | 0.6313 0.2136 | 0.4393

Access to peers/networks | 0.7818 | 0.3640

Locally applicable research | 0.7671 | 0.4385

--------------------------------------------------------------------------

(blanks represent abs(loading)<.138)

**Table S3.5-5**

**Factor loading matrix –rotated (oblique promax), 2-factors retained: Attitudes to issues needed to be addressed to improve practice**

-----------------------------------------------------------------

Variable | Factor1 Factor2 | Uniqueness

-------------+--------------------+------------------------------

Financial incentives | 0.5220 | 0.7447

More staff | 0.6697 | 0.6210

Training | 0.1804 0.5442 | 0.5619

Feedback on performance | 0.6214 | 0.5174

Equipment/supplies | 0.2117 0.5149 | 0.5686

Security | 0.4540 0.1929 | 0.6591

Physical environment | 0.3541 0.2953 | 0.6710

Higher quality of research | 0.7794 | 0.4103

Access to peers/networks | 0.8303 | 0.3741

Locally applicable research | 0.6626 | 0.5429

-----------------------------------------------------------------

(blanks represent abs(loading)<.138)
